# Asymptomatic Patient With Incarcerated Gravid Uterus Diagnosed in the Third Trimester: A Case Report of a Rare Potential Obstetric Emergency

**DOI:** 10.7759/cureus.45117

**Published:** 2023-09-12

**Authors:** Mark E Eskander, Sahejmeet S Guraya, Sohrab Afshari Mirak, Inas Mohamed

**Affiliations:** 1 Department of Radiology, Lake Erie College of Osteopathic Medicine, Erie, USA; 2 Department of Radiology, University Hospitals Cleveland Medical Center/Case Western Reserve University, Cleveland, USA

**Keywords:** classical cesarean, pelvic mri, pelvic ultrasound, oligohydramnios, cesarean section, pregnancy, obstetrics and gynecology, intrauterine growth retardation, incarcerated gravid uterus, incarcerated uterus

## Abstract

Incarcerated gravid uterus (IGU) is a rare condition that occurs when a retropositioned gravid uterus becomes entrapped within the pelvic cavity. Most patients present around the 17th week of pregnancy with symptoms such as pelvic fullness, urinary incontinence, abdominal pain, constipation, and vaginal bleeding. Rarely, patients are asymptomatic throughout pregnancy, leaving IGU undiagnosed and untreated. Here, we present an asymptomatic 26-year-old female who presented at 30 weeks of gestation with severe intrauterine growth retardation (IUGR) on serial obstetric ultrasounds. Further evaluation with ultrasound and MRI revealed an incarcerated uterus. This was complicated by severe fetal IUGR, abnormal biophysical profile, and oligohydramnios. This case highlights the importance of early diagnosis and treatment of IGU in order to prevent complications associated with the condition. Clinicians should be aware that, although uncommon, patients with IGU may be asymptomatic and that diagnosis should depend primarily on imaging findings rather than symptoms.

## Introduction

Incarcerated gravid uterus (IGU) is a rare potential obstetric emergency that occurs when a retropositioned uterus becomes entrapped in the pelvis during pregnancy. A retropositioned uterus is considered a normal variant in the first trimester; about 15% of females have a retroflexed uterus before pregnancy, and 11% of females develop a retroflexed uterus during the first trimester of pregnancy [1]. In most cases, the uterus moves up into the abdominal cavity around the 14th week of pregnancy. IGU is diagnosed when the uterine fundus becomes incarcerated between the sacral promontory and symphysis pubis and subsequently fails to ascend after the 16th week of gestation. This phenomenon is rare, occurring in only one in 3,000 pregnancies [2]. Patients are usually diagnosed around the 17th week of pregnancy when they present for evaluation of symptoms such as pelvic fullness, urinary incontinence, abdominal pain, constipation, and vaginal bleeding [3]. However, a minority of patients are asymptomatic throughout pregnancy, leading to a lack of suspicion and failure to diagnose the condition. This increases the likelihood of complications, some of which are potentially devastating. Early detection and treatment can be accomplished by relying on imaging findings rather than symptoms for diagnosis. Here, we discuss an asymptomatic 26-year-old female at 30 weeks of gestation who presented with severe intrauterine growth retardation (IUGR) detected on obstetric ultrasounds. Further evaluation revealed an incarcerated uterus and a nonreassuring fetal status. A preterm cesarean section was performed at 31 weeks and one day. We discuss the diagnoses, management, and outcomes of IGU.

## Case presentation

**Figure 1:**
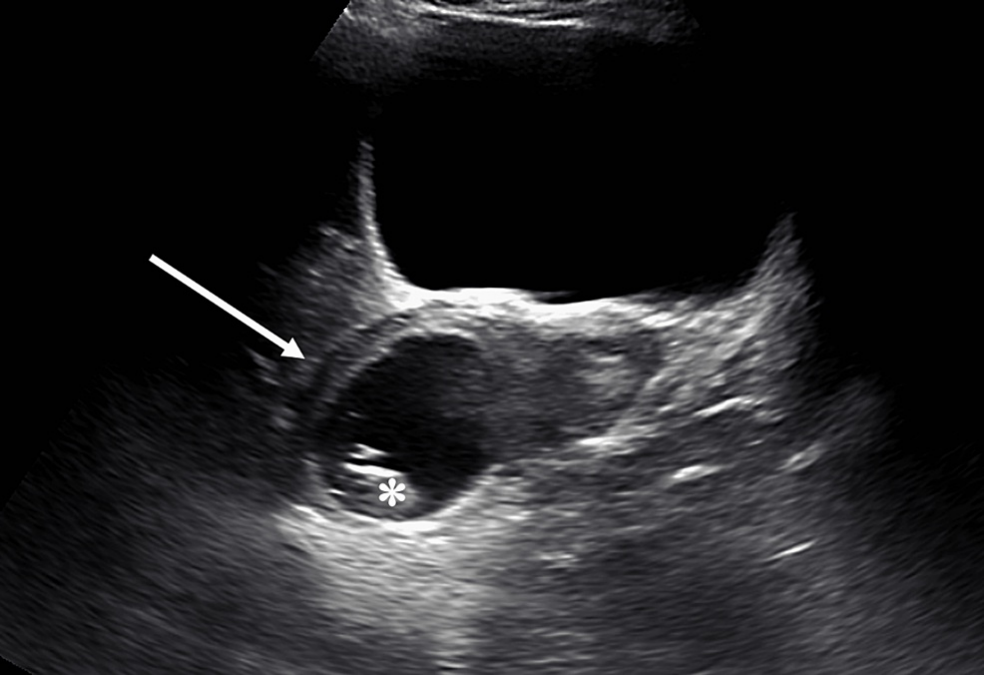
Transabdominal pelvic ultrasound long-axis view in the first trimester shows a retroverted uterus (white arrow) and intrauterine fetus (asterisk).

A 26-year-old G2 P0010 (at time of presentation) female with an estimated gestational age (EGA) of 30 weeks presented with IUGR and oligohydramnios detected on serial obstetric ultrasounds. The patient has a history of a known retroverted uterus and a prior spontaneous abortion at 10 weeks of gestation. A transabdominal pelvic ultrasound in the first trimester showed the retroverted uterus and the intrauterine fetus (Figure 1).

**Figure 2:**
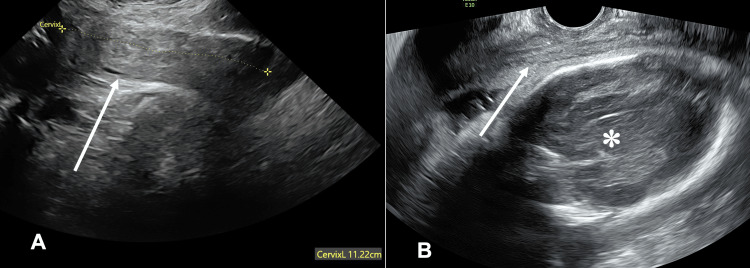
Transvaginal ultrasound long-axis view (A, B) at 30 weeks of gestation shows an elongated cervix (arrows) measuring 11.22 cm, coursing anterior to the uterus (asterisk).

The patient denied any urinary urgency, incontinence, constipation, pelvic pain, or vaginal bleeding throughout her pregnancy. A transvaginal ultrasound was performed at 30 weeks of gestation and showed a markedly elongated cervix that was displaced anteriorly and to the left of the uterus (Figures 2A-2B). The uterine fundus was not identified.

**Figure 3:**
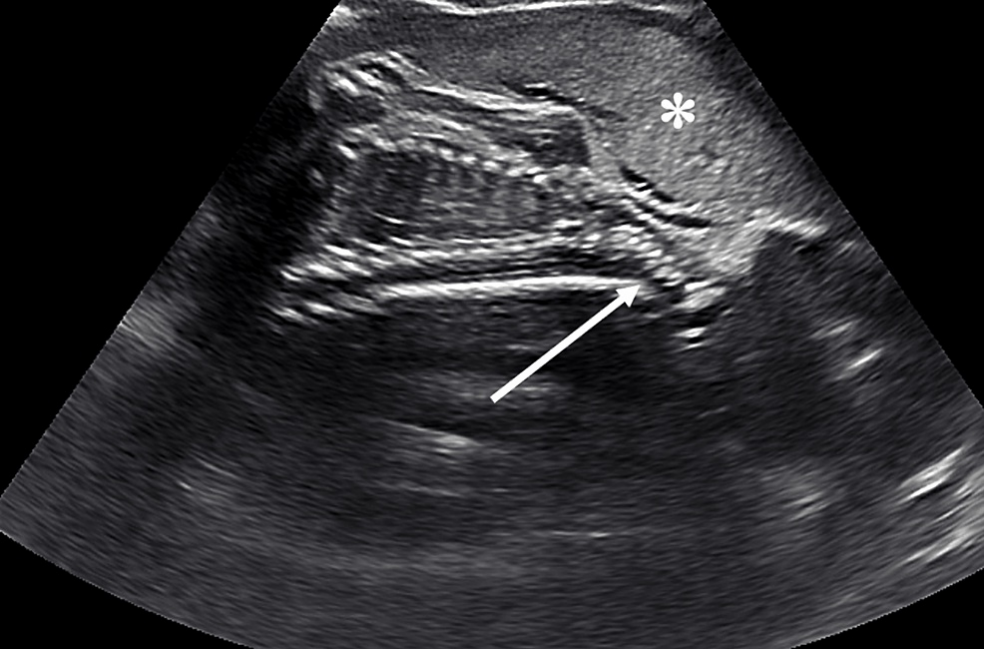
Transabdominal ultrasound long-axis view at 30 weeks of gestation shows the hyperextended neck of the fetus (arrow) with the face-up. The placenta is anterior (asterisk).

On transabdominal ultrasound, the placenta was located anteriorly without previa, and the fetal neck was hyperextended with the fetus positioned face-up (Figure 3). The estimated weight of the fetus was 946 g (< 1st percentile). The EGA by ultrasound was 26 weeks and two days (< 1st percentile). Oligohydramnios was noted with low amniotic fluid volume. No fetal anomalies were identified.

**Figure 4:**
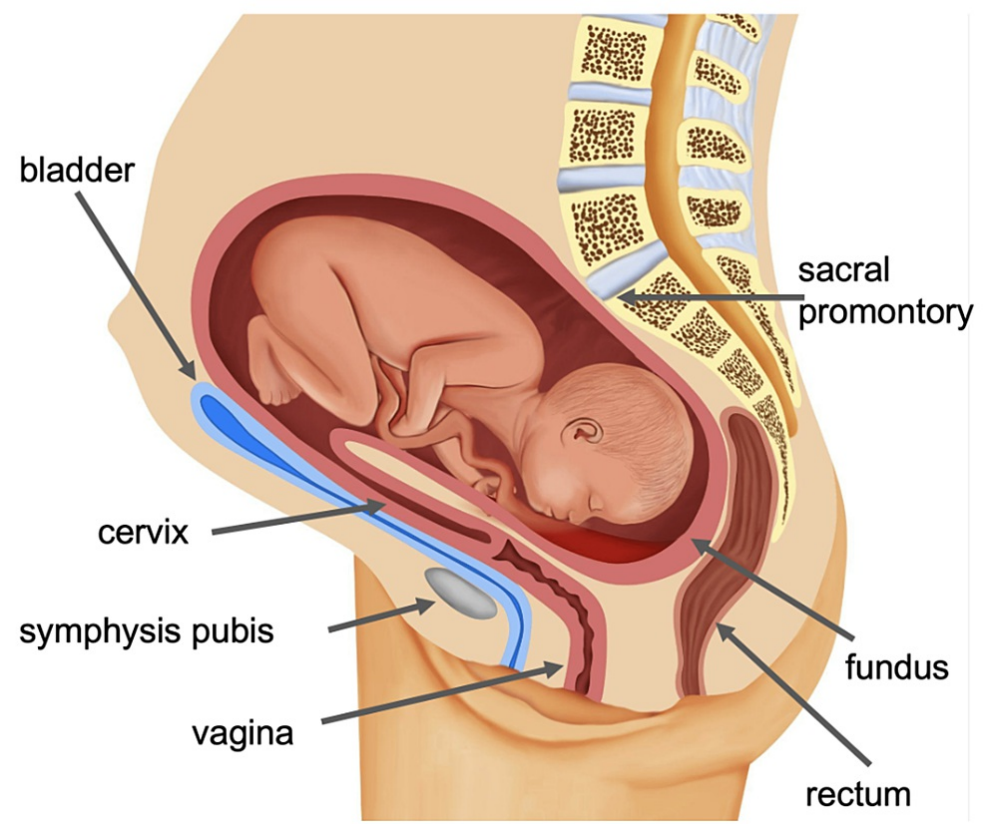
An illustration of the IGU. IGU: incarcerated gravid uterus Image credit: Inas Mohamed

**Figure 5:**
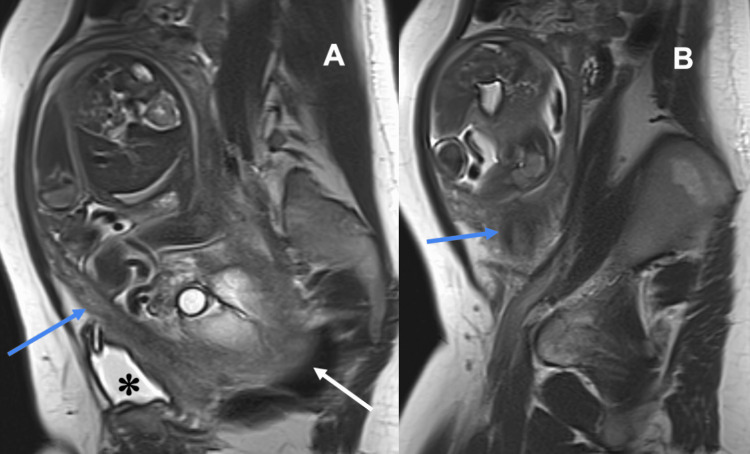
Sagittal (A, B) T2-weighted MRI images of the abdomen and pelvis without IV contrast show the markedly elongated cervix (blue arrows) coursing anterolateral to the uterus. The retropositioned uterus is entrapped in the pelvis with the fundus (white arrow) embedded in the sacrum and the bladder (asterisk) compressed anterior to the uterus.

**Figure 6:**
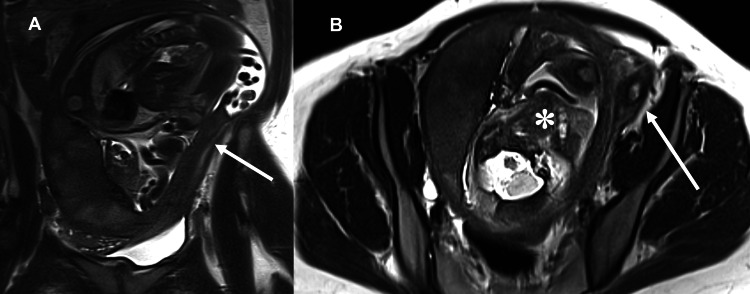
Coronal (A) and axial (B) T2-weighted MRI images of the abdomen and pelvis without IV contrast show the markedly elongated cervix (arrow) coursing to the left of the retropositioned uterus (asterisk).

A spin tomography in time-domain (STAT) MRI of the abdomen and pelvis without IV contrast was performed. The uterus was significantly retropositioned, with the inverted fundus entrapped in the pelvis and embedded in the sacrum below the promontory. The vagina and cervix were markedly elongated, with the stretched cervix coursing anteriorly and to the left of the uterus. The cervical internal os was proximal to the urinary bladder, which was compressed anterior to the uterus. The fetal head was in the fundus in a complete breech presentation. The findings were compatible with an incarcerated gravid uterus (Figures 4-6B).

**Figure 7:**
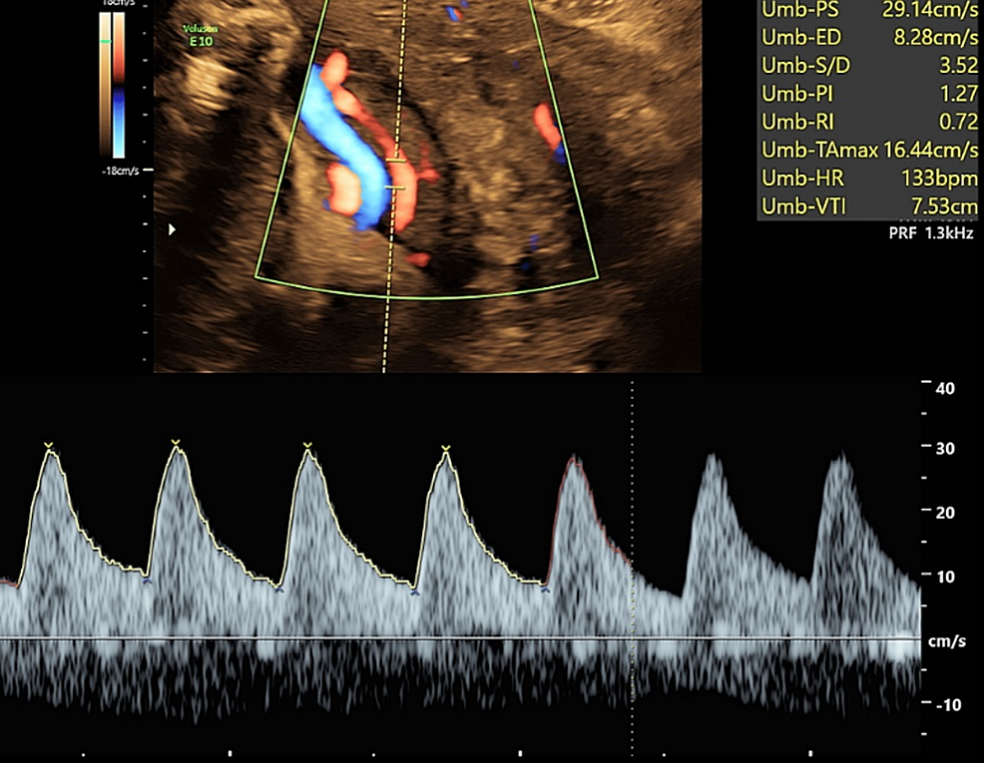
Doppler ultrasound of the umbilical artery of the fetus at 31 weeks of gestation shows elevated SDR at 3.52 (94%), PI at 1.27 (91%), and RI at 0.72 (86%). SDR: systolic velocity/diastolic velocity ratio PI: pulsatility index RI: resistive index

**Figure 8:**
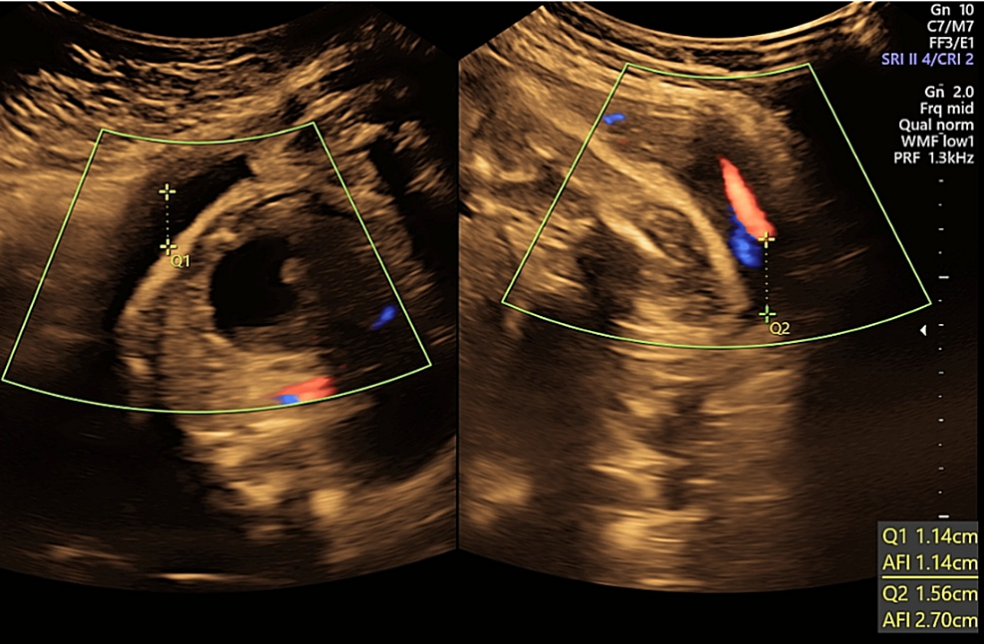
Transabdominal pelvic ultrasound at 31 weeks of gestation shows oligohydramnios with two small pockets of amniotic fluid and an estimated AFI of 3.8 cm. AFI: amniotic fluid index

The patient subsequently underwent a targeted growth ultrasound that included a fetal biophysical profile with a non-stress test (NST) and an umbilical artery Doppler. The umbilical artery systolic velocity/diastolic velocity ratio (SDR), pulsatility index (PI), and resistive index (RI) were all elevated at 94%, 91%, and 86%, respectively (Figure 7). The amniotic fluid index (AFI) was low (3.8) (Figure 8). The fetal biophysical profile was abnormal, with a score of 6/8 due to the presence of oligohydramnios. The fetal NST was also abnormal, demonstrating recurrent prolonged decelerations.

The clinical decision was made to perform an unscheduled, non-urgent cesarean section for nonreassuring fetal status in the setting of a known incarcerated uterus. The patient received two doses of betamethasone injections prior to delivery. A cesarean section was performed at an EGA of 31 weeks and one day under spinal anesthesia. To avoid injuring the distorted bladder, cervix, and vagina, a vertical midline incision was made. Through electrocautery and stretching, the fascia was incised in the midline and extended cephalocaudally. The rectus muscle was divided bluntly in the midline. The peritoneum was entered bluntly, taking care to avoid injuring the viscera. The uterus was grasped, and the fundus was gently lifted from the pelvis and reoriented into its normal anatomical position. A classical uterine incision was performed. The bladder blade was inserted. The premature infant was delivered atraumatically and was handed off to an awaiting neonatal intensive care unit (NICU) nurse. The birth weight was 2 lbs, 14 oz. The newborn complications included respiratory distress and NICU admission. There were no immediate postoperative maternal complications. Both the mother and the neonate were later discharged from the hospital in satisfactory condition. The patient was advised to have a preconception consultation with maternal-fetal medicine to manage any future pregnancies. Due to her classical cesarean section, she would require a cesarean delivery for all future pregnancies as a vaginal birth would increase the risk of uterine rupture. The cesarean section would likely be planned for 36-37 weeks of gestation.

## Discussion

IGU is a rare condition that occurs when the gravid uterus is entrapped in the pelvic cavity between the sacral promontory and pubic symphysis past 16 weeks of gestation. Risk factors for the condition include a retroverted or retroflexed uterus, endometriosis, leiomyomas, uterine anomalies, pelvic adhesions, previous abdominopelvic surgery, a deep sacral concavity with an overlying sacral promontory, and an incarcerated uterus in a previous pregnancy [4].

Most patients with IGU present in the second trimester with symptoms due to compression of structures around the incarcerated uterus, such as urinary manifestations, abdominal pain, constipation, and vaginal bleeding. However, IGU may rarely be asymptomatic and thus may not be detected during regular prenatal visits due to a lack of suspicion. In such cases, IGU may not be diagnosed until the third trimester, when further evaluation is performed due to the presence of fetal complications such as IUGR, which was the case for our patient. Thus, early diagnosis should be based primarily on imaging findings rather than clinical symptoms. On transvaginal ultrasound, the cervix is elongated, anteriorly displaced, and stretches upward, superior to the bladder and pubic symphysis. This may make the cervix difficult to identify on ultrasound. Thus, difficulty identifying the cervix on ultrasound, especially in patients with predisposing factors, should raise suspicion for IGU and prompt further investigation. Of note, due to the abnormal configuration of the cervix, it may be confused as an empty uterus on ultrasound; this may result in misdiagnosing IGU as an ectopic pregnancy. A retroverted or retroflexed uterus may also be visualized. Importantly, an MRI is essential to confirm the diagnoses as it provides excellent visualization of the anatomic relationships in the pelvis, which is necessary for diagnosis. The uterus will be retropositioned, with the fundus embedded in the hollow of the sacrum. The cervix will be elongated and displaced anteriorly relative to the uterus. The bladder will be displaced superior to the pubic symphysis and compressed anterior to the uterus. Pelvic exam findings are also useful for diagnosis. On a pelvic exam, the cervix is difficult to expose due to its anterior and superior displacement against the pubic symphysis. Sacculation of the posterior wall of the vagina and the posterior fornix bulge may also be observed. The uterine fundus may be immobile and palpated within the curvature of the sacrum.

The complications of IGU can be devastating. Serious maternal complications include bladder rupture, renal failure, uterine ischemia, uterine rupture, premature labor and delivery, and injury of the bladder, cervix, and vagina during delivery. Serious fetal complications include miscarriage in the first trimester, fetal death, and IUGR [5].

The initial management of IGU includes attempting to reduce the incarcerated uterus through urinary bladder drainage, followed by chest-knee maneuvers. If this fails, manual uterine reduction may be attempted. Manual reduction poses serious risks, such as placental abruption, rupture of membranes, intrauterine fetal death, and preterm delivery. Manual reduction is only recommended before 20 weeks of gestation, as the risk of preterm birth is higher when performed beyond this point. In cases where patients are diagnosed past 20 weeks of gestation or if manual reduction fails, careful follow-up until a planned cesarean section is the preferred route of management. A cesarean section is necessary because the incarcerated uterus may impair the descent of the fetus. A vertical supraumbilical approach should be taken in order to avoid injuring the bladder, cervix, and vagina [6]. At any point in pregnancy, expectant management with close follow-up is also an option, as spontaneous resolution of the incarcerated uterus is common. In one retrospective study of 14 patients with IGU, 11 (78.5%) patients had spontaneous resolution of the incarcerated uterus after 16 weeks of gestation [3].

Of note, there is a lack of general consensus regarding the optimal management of IGU due to the rarity of the condition. Considering the reasonable probability that IGU may resolve spontaneously and that manual reduction poses a risk of serious complications, some authors question if manual reduction is justified over expectant management, especially in patients without severe symptoms due to incarceration [3]. In our case, early diagnosis and treatment would have been preferred, despite the patient being asymptomatic. This is based on the outcome of severe IUGR and fetal distress, which were complications of the undiagnosed and untreated incarcerated uterus.

## Conclusions

Despite being exceedingly rare, it is important for clinicians to be aware of the possibility that patients with IGU will remain asymptomatic throughout pregnancy. Thus, imaging and pelvic exam findings should be relied on for early diagnoses of the condition. This allows for timely treatment and prevention of the potentially devastating complications of IGU.
